# Supplementary material for: The cross-cultural validation of the Beach Center Family Quality of Life Scale among persons affected by leprosy or podoconiosis in Northwest Ethiopia
Source: PLoS Negl Trop Dis. 2023 Oct 6;17(10):e0011235. doi: 10.1371/journal.pntd.0011235 (PMC10584103; doi:10.1371/journal.pntd.0011235)
Supplement: S1 STROBE — (DOCX) [file pntd.0011235.s001.docx]

STROBE Statement—Checklist of items that should be included in reports of ***cross-sectional studies***

**“****The cross-cultural validation of the Beach Center Family Quality of Life Scale among persons affected by leprosy and podoconiosis in Northwest, Ethiopia”**

|  | Item No | Recommendation |
| --- | --- | --- |
| **Title and abstract** | 1 | (*a*) Indicate the study’s design with a commonly used term in the title or the abstract Abstract, paragraph 2 (under heading methodology). |
|  |  | (*b*) Provide in the abstract an informative and balanced summary of what was done and what was found Abstract, paragraphs 2 and 3 (under the headings methodology and results). |
| Introduction | | |
| Background/rationale | 2 | Explain the scientific background and rationale for the investigation being reported Introduction, all paragraphs; the third and fourth paragraphs specifically. |
| Objectives | 3 | State-specific objectives, including any prespecified hypotheses Introduction, last paragraph |
| Methods | | |
| Study design | 4 | Present key elements of study design early in the paper Methodology, sub-heading study design. |
| Setting | 5 | Describe the setting, locations, and relevant dates, including periods of recruitment, exposure, follow-up, and data collection Methodology, sub-heading study site, and relevant dates of data collection can be found under the data collection sub-heading. |
| Participants | 6 | (*a*) Give the eligibility criteria and the sources and methods of selection of participants Methodology, sub-headings study population, sample size, sampling technique, and eligibility criteria. |
| Variables | 7 | Clearly define all outcomes, exposures, predictors, potential confounders, and effect modifiers. Give diagnostic criteria, if applicable Methodology, sub-heading measures, and data collection. |
| Data sources/ measurement | 8* | For each variable of interest, give sources of data and details of methods of assessment (measurement). Describe the comparability of assessment methods if there is more than one group Methodology, sub-headings measurement, data collection. |
| Bias | 9 | Describe any efforts to address potential sources of bias Methodology, sub-headings data collection, and data analysis. |
| Study size | 10 | Explain how the study size was arrived at Methodology, sub-heading study population, sample size, and sampling technique. |
| Quantitative variables | 11 | Explain how quantitative variables were handled in the analyses. If applicable, describe which groupings were chosen and why Methodology, sub-heading data analysis. |
| Statistical methods | 12 | (*a*) Describe all statistical methods, including those used to control for confounding Methodology, sub-heading data analysis. |
|  |  | (*b*) Describe any methods used to examine subgroups and interactions Methodology, sub-heading data analysis. |
|  |  | (*c*) Explain how missing data were addressed Not available. |
|  |  | (*d*) If applicable, describe analytical methods taking account of the sampling strategy Not available. |
|  |  | (*e*) Describe any sensitivity analyses Not available. |
| Results | | |
| Participants | 13* | (a) Report numbers of individuals at each stage of study—eg numbers potentially eligible, examined for eligibility, confirmed eligible, included in the study, completing follow-up, and analysed Methodology, sub-heading translation, and adaptation process,  Results, sub-heading, socio-demographic characteristics of the study participants**,** Table 1. |
|  |  | (b) Give reasons for non-participation at each stage Not available. |
|  |  | (c) Consider the use of a flow diagram We have used tables and figures: please see the ‘results’ heading. |
| Descriptive data | 14* | (a) Give characteristics of study participants (eg demographic, clinical, social) and information on exposures and potential confounders Results, sub-heading socio-demographic demographic characteristics of the study participants**,** Table 1. |
|  |  | (b) Indicate the number of participants with missing data for each variable of interest Not available. |
| Outcome data | 15* | Report numbers of outcome events or summary measures Results indicate the outcomes measured. |
| Main results | 16 | (*a*) Give unadjusted estimates and, if applicable, confounder-adjusted estimates and their precision (eg, 95% confidence interval). Make clear which confounders were adjusted for and why they were included Results, sub-heading reproducibility: reliability and agreement, Table 2. |
|  |  | (*b*) Report category boundaries when continuous variables were categorized Results, sub-heading socio-demographic characteristics of the study participants, description part, and Table 1. |
|  |  | (*c*) If relevant, consider translating estimates of relative risk into absolute risk for a meaningful time period Not available. |
| Other analyses | 17 | Report other analyses done—eg analyses of subgroups and interactions and sensitivity analyses Results, ‘Interpretability’ subheading. |
| Discussion | | |
| Key results | 18 | Summarise key results with reference to study objectives Discussion paragraphs 2-6. |
| Limitations | 19 | Discuss the limitations of the study, taking into account sources of potential bias or imprecision. Discuss both the direction and magnitude of any potential bias Discussion, last paragraph. |
| Interpretation | 20 | Give a cautious overall interpretation of results considering objectives, limitations, the multiplicity of analyses, results from similar studies, and other relevant evidence Discussion, conclusion, and recommendation headings. |
| Generalisability | 21 | Discuss the generalisability (external validity) of the study results Discussion, conclusion, and recommendations headings. |
| Other information | | |
| Funding | 22 | Give the source of funding and the role of the funders for the present study and, if applicable, for the original study on which the present article is based The sub-heading Funding. |

*Give information separately for exposed and unexposed groups.
