# Supplementary material for: The cross-cultural validation of the Beach Center Family Quality of Life Scale among persons affected by leprosy or podoconiosis in Northwest Ethiopia
Source: PLoS Negl Trop Dis. 2023 Oct 6;17(10):e0011235. doi: 10.1371/journal.pntd.0011235 (PMC10584103; doi:10.1371/journal.pntd.0011235)
Supplement: S1 Table — (DOCX) [file pntd.0011235.s002.docx]

**BACK-TRANSLATION REVIEW**

| ***The back-translation Sufficiency rating shall be done based on the following two parameters:*** | |
| --- | --- |
| ***Semantic equivalence:*** | **Does the expression of the translated item achieve the same literal meaning as the source item?**  **Do the words mean the same thing?**  **Are there multiple meanings of the word in the back translated item that might create different response sets by respondents (if used in the source population)?**  **Are there grammatical difficulties/problems in the translation?** |
| ***Conceptual equivalence:*** | **Do the back-translated items adequately describe the concept in the source language and adequately map to the intended purpose of the item? Please note that often words hold different conceptual meanings between cultures and different socio-demographic and economic groups.** |

| ***Form 1: FQoL 25 Question Family Quality of Life Indicator to measure the affected person and family members towards persons with Leprosy and podoconiosis.*** |
| --- |
| ***Sufficiency (translation quality) Ratings (Please refer to the above two sufficiency parameter definitions above):***  1= Inadequate (needs detailed special review, describe nature of the problem)  2= Questionable (needs discussion, describe nature of the problem)  3= Adequate (no comment needed) |

**Family Quality of Life in Ethiopian context**

| No | **Source document** | **Translation 1** | Sufficiency rating 1 | Comment | **Translation 2** | Sufficiency rating 1 | Comment |
| --- | --- | --- | --- | --- | --- | --- | --- |
| 1 | My family enjoys spending time together. | My family is happy about the time they spend together | 3 |  | My family is happy in spending time together. | 3 |  |
| 2 | My family members help the children learn to be independent. | My family members support children to be self-dependent and responsible. | 3 |  | My family members help children to know about self-reliance | 3 |  |
| 3 | My family has the support we need to relieve stress. | My family members have support for getting out of worry/stress. | 3 |  | My family members have support to cope with stress. | 3 |  |
| 4 | My family members have friends or others who provide support. | My family members have friends or other people to support them | 3 |  | My family members have friends or people to provide support. | 3 |  |
| 5 | My family members help the children with schoolwork and activities. | My family members support children with schoolwork and similar activities | 3 |  | My family members support children in their school work and related tasks. | 3 | “Activities” in the U.S. refers to school-sponsored sports and clubs. If your country doesn’t have this, then “support children on school work” should be sufficient. |
| 6 | My family members have transportation to get to the places, they need to be. | My family members have transportation to go to any place they want to go to | 3 |  | My family members have transportation to go to places they want. | 3 |  |
| 7 | My family members talk openly with each other. | My family members openly discuss any issue | 3 |  | My family members make open discussions about everything. | 3 |  |
| 8 | My family members teach the children how to get along with others. | My family members teach children how they can peacefully live with other people | 3 |  | My family members teach children how to live or agree with people. | 3 |  |
| 9 | My family members have some time to pursue our own interests. | My family members give us some time to satisfy our needs | 2 | Satisfy our needs may not replace pursue our own interests. | My family members give us some time to fulfill our interests. | 3 | This is probably the better version of this item. |
| 10 | Our family solves problems together. | My family members solve their problems together | 3 |  | My family members solve their problem | 2 | Is not showing their togetherness |
| 11 | My family members support each other to accomplish goals. | My family members cooperate with each other to manage their life | 2 | Manage their life may not be similar with accomplish goals | My family members help each other to be successful in their lives. | 3 | Better |
| 12 | My family members show that they love and  care for each other. | My family members show love and cooperation. | 2 | Cooperation is looser than caring for each other. | My family members show love and care for each other | 3 |  |
| 13 | My family has outside help available to us to take care of the special needs of all family members. | There is another body/person to support my family in times of difficulty | 1 | In times of difficulty does not replace the special need | We have another person or person to take care of the special needs of my family members | 3 |  |
| 14 | Adults in our family teach the children to make good decisions. | Adults in our family teach the children to make good decisions | 3 | Delete the wise person | Elders and adults in the family teach children to make good decisions. | 3 |  |
| 15 | My family gets medical care when needed. | The family members get medical support when necessary | 3 |  | Members of the family get medical support when it is necessary. | 3 |  |
| 16 | My family has a way to take care of our expenses. | My family has a way to cover the expenses to take care of ourselves | 3 |  | My family has the means to cover the expenses to take care of ourselves | 3 |  |
| 17 | Adults in my family know other people in the children’s lives (friends, teachers, etc.). | Adult members in my family know the lives of other people in relation to their children’s life (friends, teachers, etc.) | 3 |  | Elders of My family members know other people (friends, teachers etc.) who are in the lives of their children. | 3 |  |
| 18 | My family is able to handle life’s ups and downs. | My family members can overcome the ups and downs of life. | 3 | This is better. | My family members can stand lives ups and downs. | 3 |  |
| 19 | Adults in my family have time to take care of the individual needs of every child. | Adults in my family have time to satisfy the interest of each child | 3 |  | Adults of my family members have time to fulfill the individual interest of every child. | 3 |  |
| 20 | My family gets dental care when needed. | My family gets dental treatment when necessary | 3 |  | My family members can get dental medication when it is necessary. | 3 | The medication changed to treatment |
| 21 | My family feels safe at home, work, school, and in our neighborhood. | My family feels safe at home, in the workplace, at school, and in the neighborhood. | 3 |  | My family members feel secure at home, workplace, school, and neighborhood. | 3 |  |
| 22 | My family member with a disability has support to accomplish goals at school or at workplace. | A family member with a disability gets appropriate support to achieve his goal at school or in the workplace. | 3 | Delete appropriate | Disabled members of my family have proper support to fulfill their goals at the workplace or school. | 3 | Delete proper |
| 23 | My family member with a disability has support to accomplish goals at home. | A family member with a disability gets support at home to succeed in his goal | 3 | His change to their | Disabled members of my family have support to fulfill their goals at home. | 3 |  |
| 24 | My family member with a disability has support to make friends. | A family member with a disability is encouraged to have his/her own friends | 3 |  | A disabled member of my family is motivated to have friends. | 3 |  |
| 25 | My family has good relationships with the service providers who provide services and support to our family members with a disability. | My family members have a good relationship with people who provide support and service for the disabled family member | 3 |  | My family members have good relationships with people who give service and support to disabled members of my family. | 3 |  |

Comment by original scale developer

In general, I agree with the other reviewers. I have inserted a couple of comments to clarify some of the items.

On items 21 through 25 (supports for the family member with a disability), I see nothing wrong with making those items specific to people who are experiencing the particular disabilities in your study. I do not know what kinds of services are offered to people with these conditions in Ethiopia, but perhaps the respondents would understand better how to answer if you made those items specific to the disabilities and used the names of the services in your country. I know of others who used this scale who have done this.
